# Collectivism-individualism: Strategic behavior in tacit coordination games

**DOI:** 10.1371/journal.pone.0226929

**Published:** 2020-02-04

**Authors:** Dor Mizrahi, Ilan Laufer, Inon Zuckerman

**Affiliations:** Department of Industrial Engineering and Management, Ariel University, Ariel, Israel; Middlesex University, UNITED KINGDOM

## Abstract

The effect of culture on strategic interaction has been widely explored. However, the effect of the cultural background on focal point selection in tacit coordination games has not yet been examined. To accomplish this goal, in this study we have focused on the individual level of analysis. That is, we constructed a strategic profile to model the behavior of each individual player and then used unsupervised learning methods on the individual data points. We have chosen to examine two groups of participants, Israelis (ICB) and Chinese (CCB), each belonging to a different cultural background representing individualist and collectivist societies, respectively. Clustering the individual strategic profiles has allowed us to gain further insights regarding the differences between the behavioral strategies of each cultural group. The results of this study demonstrate that the cultural background has a profound effect on the strategic profile and on the ability to succeed in tacit coordination games. Moreover, the current study emphasizes the importance of relying on the individual level of analysis and not only on the group level of analysis. The implications of these results and potential future studies are discussed.

## Introduction

The effect of culture on strategic interaction has been widely explored [1–8]. However, the effect of culture on focal point selection in tacit coordination games has not yet been examined. Tacit coordination games are coordination games in which communication between the players is not allowed or not possible [2]. As has been shown in many studies (e.g. [3,9–11]), people are able to coordinate sometimes much better than predicted by game theory by somehow converging to a set of prominent solutions called focal points. In tacit coordination games all the solutions are associated with an equal payoff to both players, and therefore the only task of the players is to converge on the same solution. That solution is denoted as a focal point. In contrast, other economic games (e.g., Ultimatum game, Dictator, battle of the sexes, prisoner’s dilemma) all include a strategic element associated with the division of goods resulting in that players might receive asymmetric payoffs for different solutions.

Previously, it has been shown that culture affects the mental model (or schemas) of the players such that players that belong to the same culture share similar preconceptions and belief systems that impact their strategic thinking (e.g. [12–14]). For example, in [15] the authors showed that the cultural background (either American or Indian) had an effect on both the frequency of coordination and the payoff level in a variation of a battle of the sexes game. Recently, the effect of the cultural background on the distribution of resources between players and an unknown recipient was demonstrated in the context of tacit coordination games [16]. In contrast, our study has not involved either asymmetric payoffs or distribution of resources and focused on investigating the effect of the cultural background on focal point selection per se.

Moreover, there are two substantial differences between our study and previous studies. First, previously, examination of the effect of culture on strategic interactions has relied on the group level of analysis [15]. That is, the measures in these studies were aggregated across individual players and were analyzed as such. For example, in [15] the comparison between the cultures was based on the frequency of choosing certain actions which was treated as a group-level (or aggregated) measure. Likewise, in [10] the coordination index (CI) of each game was computed based on the aggregated answers across all the participants playing that game. In contrast, in this study the emphasis was on the individual level of analysis. That is, we constructed a strategic profile to model the behavior of each individual player and then used unsupervised learning methods on the individual data points. Second, in previous studies the game instances that have been used mostly relied on selecting a single action (e.g. [3,11,15]). However, in the current study the individual strategic model that we have constructed was based on data collected across multiple game instances whose complexity level was higher since the player had to make several decisions in each of them.

To implement these two key points in our study, we have constructed for each player an individual strategic profile comprising the individual propensities to apply different selection rules. Then, we have clustered these individual strategic profiles to examine the differences in implementing prominent strategies between two different cultures. Thus, the individual level of analysis enabled us to demonstrate, for the first time, the existing variability in strategic behavior between players belonging to two different cultures in the context of assessing tacit coordination ability. This was previously not possible because studies have relied on aggregated group data and therefore information regarding the differences at the level of the individual player was lost. In addition, we demonstrated the importance of knowing the identity of the cultural background for improving the accuracy of predicting the coordination ability at the individual level.

We have chosen to examine two groups of participants, Israelis (ICB) and Chinese (CCB), each belonging to a different cultural background representing individualist and collectivist societies, respectively. The Individualism vs. Collectivism dimension (denoted as I-C) is highly explored in the scientific literature (e.g. [10,11,16,17]). While the Chinese society is considered to be a good exemplar of a collectivist society [18,19], the Israeli society is considered to be a mix of individualistic and Collectivist cultures [11,16]. Hence, in this study we aimed to examine the effect of the cultural differences on the variability of individual strategic behavior within each of the cultural groups in the context of focal point selection.

## Materials and methods

### Experimental design

**Fig 1:**
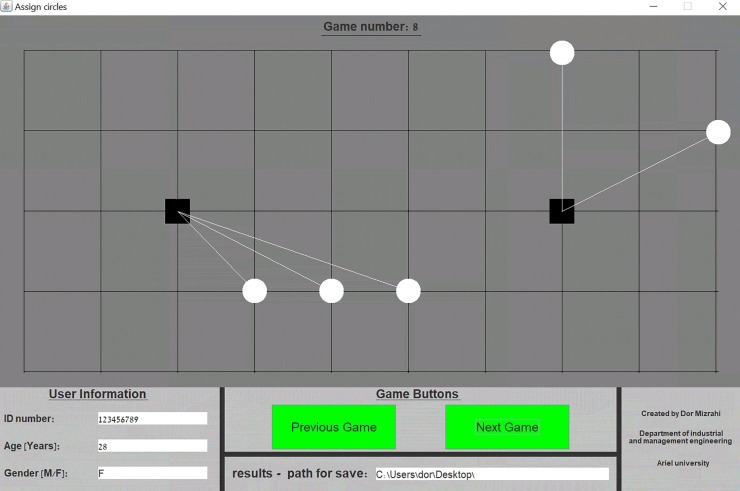
"Assign Circles" application window.

To test the coordination ability of players we used the "Assign Circles" [3,10] tacit coordination game. This is a board game in which players are asked to assign circles to squares with the aim of coordinating their assignment with an unknown player who is presented with the same board (see Fig 1 for a game example). That is, a successful coordination is achieved when both players attach all the circles to the same squares. In case of a successful coordination both players gain a point and the score is accumulated as the game progresses. There are two main parameters that impact the coordination difficulty level of a game: the number of circles and the positions of the circles on the board. The players were presented in total with 14 different "Assign Circles" board games: 10 games were taken from [3] (See Supporting Information–S1 Appendix) and therefore their parameters were already predefined. In four additional games the number of circles (~ Uniform[1, 7]) and their placement were randomized for each player by the computer. Participants were told that they would earn bonus points that will be added to the final course grade according to the total number of points that was accumulated. Both players had no communication capability at all, and their total gained score was only revealed after all the games have been completed (so no feedback was provided between game instances). The instructions to the participants are given in Supporting Information–S1 Appendix.

### Participants and procedure

The participants were composed of two different groups that were involved in two different sessions, respectively. The first group contained 93 students from Ariel University that were enrolled in one of the courses on campus (49 of whom were female, 83 were right hand dominant, mean age = ~ 23, SD = 1.97). The second group contained 95 students at the University of Chinese Academy of Sciences (UCAS) that were enrolled in one of the courses on campus (36 of whom were female, 88 were right hand dominant, mean age = ~ 23, SD = 1.89). The study was approved by the IRB committee of Ariel University and the University of Chinese Academy of Sciences (UCAS) from which students were randomly drawn as participants in the study. All the participants provided written informed consent for participating in the experiment.

The experiment was carried out in the same manner for each of the two groups. First, the participants received an explanation regarding the experimental procedure and the game application. Next, they read an additional written instructions file (see Supporting Information–S1 Appendix), filled out their age and gender, and participated in the "Assign Circles" game. Each of the participants played 14 different board games (in a random order). Finally, the output file of each experiment was uploaded to a shared location for offline analysis. After collecting the data from both sessions, participants were randomly divided into dyads. That is, each participant was paired with a dyad partner randomly selected from the same cultural group. The participants were aware of the fact that all other participants were from the same university.

### Measures

#### Individual coordination ability (ICA)

The individual coordination ability (ICA) reflects the individual ability of a player to succeed in tacit coordination games. The ICA index is defined as the average number of times a participant was successful in coordinating with an unknown counterpart within a set of games (alternatively, the calculation can be performed on a single game). The ICA is calculated as follows:

$$ICA(i)=\sum_{j=1|(j\neq i)}^{N}\sum_{k=1}^{t}\frac{CF(i,j,k)}{(N-1)*t}$$ (Eq 1.1)

Where i denotes the i^th^ participant, *N* is the total number of participants in the experiment, and *t* is the number of games in the experiment.

The CF (Coordination Function) is defined as follows:

$$CF(i,j,k)=\left\{ \begin{array}{l} 1;if\;players\;i\;and\;j\;chose\;the\;same\;label\;in\;game\;k\;\\ 0;else\; \end{array}\;\right.$$ (Eq 1.2)

The ICA reflects the probability of successful coordination at the level of the individual player within the group. Therefore, the ICA computation was based on the 10 predefined games only and did not include the 4 randomized games (see sub-section "experimental design") since randomization might produce varying levels of coordination difficulty among different players. Since each player played the exact same set of 10 predefined games, the ICA could be used to compare the coordination ability among players.

#### Strategy rate (SR)

In The "Assign Circles" game there are three prominent selection rules as were defined in [3,10]: (1) the rule of *closeness*–a tendency to assign each circle to the closest available square; (2) the rule of *accession*–a tendency to assign circles, which are close to one another, to the same square. Specifically, this rule is applied by first forming coherent groups according to their proximity and subsequently applying closeness on these newly created groups; (3) the rule of *equality*–a tendency to equate the number of circles that are assigned to each of the two available squares separated by an imagery vertical line such that an equal number of circles lies on the opposite sides of the line. In [3,10] it was found that players selected the focal points based on the abovementioned rules.

Since the purpose of this study was to examine the effect of cultural differences on the variability of individual strategic behavior, we have relied on a fixed and a well-defined set of selection rules as was proposed by [3,10]. Thus, here we have not aimed to re-evaluate the validity of these rules but only to utilize them as is. The complete set of the predefined games with their corresponding available selection rules is visually displayed in Supporting Information–S1 Appendix. The detailed definition of each one of the selection rules as was predetermined by [3,10] and their implementation in each of the games is presented in Supporting Information–S1 Appendix.

In this study we defined a Strategy Rate (SR) measure which reflects the probability of using each selection rule while playing the "Assigns Circles" games. This measure reflects the probability of using a specific selection rule by a specific player, the i^th^ player, based on the behavioral performance data. To compute the SR, we first define a Game Tag (GT) variable for each strategy, which can take one of three different values as follows:

$${GT(i,k)}_{Closeness\backslash Accession\backslash Equality}=\left\{ \begin{array}{c} 1\;if\;the\;strategy\;was\;available\;in\;the\;k^{th}\;game\;and\;the{\;i}^{th}\;player\;used\;it\;\\ 0\;if\;the\;strategy\;was\;not\;available\;in\;the\;k^{th}\;game\;\\ -1\;if\;the\;strategy\;was\;available\;in\;the\;k^{th}\;game\;and\;the{\;i}^{th}\;player\;didn^{\prime }t\;use\;it\; \end{array}\;\right.$$ (Eq 2.1)

The SR for each of the three selection rules can then be calculated as follows:

$${SR(i)}_{Strategy}=\frac{\sum_{k=1}^{14}\left[{GT(i,k)}_{Strategy}=1\right]}{\sum_{k=1}^{14}\left|{GT(i,k)}_{Strategy}\right|}$$ (Eq 2.2)

For player #7, for example, the number of games in which each of the strategies (Closeness, Accession, and Equality) was applicable was 8, 10, 7, respectively, and the number of games in which each of the strategies was implemented was 5, 4, and 4, respectively. Hence, the corresponding obtained SR values were as follows: 5/8 = 0.63; 4/10 = 0.40; 4/7 = 0.57.

#### Strategic profile

For each individual subject we can now define an individual *strategic profile* by weighing the propensity to implement each of the selection rules as part of the player’s decision process. Using the example of player #7 above, the player’s strategic profile is the following: {0.63, 0.4, 0.57}.

The three SR values (Closeness, Equality, and Accession) describe the specific location of each strategic profile in the participant's theoretical 3D strategic space. Based on these scores, we may then use cluster analysis of the individual strategic profiles, separately within each cultural group, and subsequently gain insights regarding the predominant strategies that were used.

## Experimental results

### Group level of analysis

We computed the grand average ICA score of both groups (ICB and CCB) across all ten games (the four randomized games were not included in the calculation of the ICA since they vary among players). Interestingly, the grand average of the Israeli group was 0.5207 while that of the Chinese group was 0.5204, thus apparently there was no difference between the groups. However, looking separately at each individual game (see Table 1) it is evident that the Chinese group was superior to the Israeli group in their coordination ability. We used the two-sample Kolmogorov-Smirnov test [20] to compare between the ICA levels of the CCB and ICB groups. Since there are ten different comparisons, we used the Bonferroni correction [21,22]. Table 1 displays the comparison between the CCB and ICB groups across the sequence of ten games. It can be seen that the CCB group outperformed the ICB group in seven out of the ten games.

**Table 1:** Statistical significance of CA values in different games as function of CB.

Game number (ICA mean values)	1	2^a^	3	4	5	6	7	8	9	10
[${\bar{ICA}}_{ICB},{\bar{ICA}}_{CCB}$]	[0.710,0.786]	[0.758,0.762]	[0.361,0.414]	[0.439,0.473]	[0.558, 0.627]	[0.526, 0.418]	[0.741, 0.494]	[0.361, 0.387]	[0.337, 0.477]	[0.416, 0.366]

(^a^—All ICA distributions are statistically significant at P<0.05, except for game #2)

**Fig 2:**
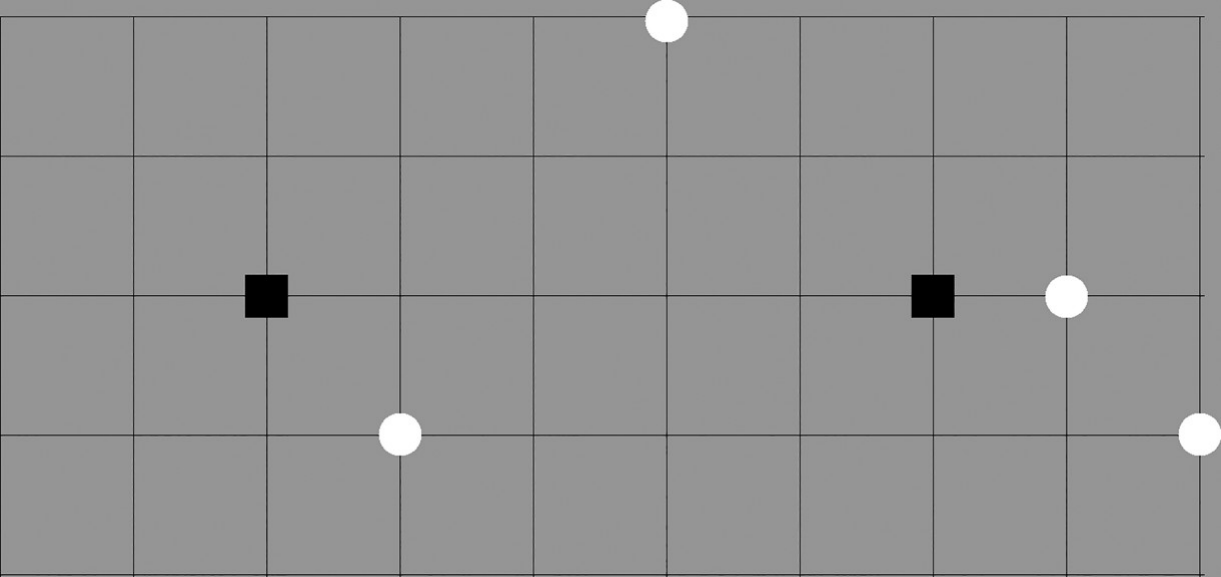
"Assign Circles"–Game #9.

Noteworthily, the results highlight the differences between the cultures that were obtained only by using a coordination ability measure that was calculated separately for each individual game. To illustrate the difference between the ICB and CCB groups in the level of coordination difficulty we chose to focus on a specific game instance (#9; The full list of games and their corresponding number can be found in Supporting Information–S1 Appendix and in [3]). In this game, a single circle splits in half the distance between two squares, as shown in Fig 2:

While the average ICA value for the ICB group was 0.337, the value of the CCB group was about 0.477. This means that while the Chinese players managed to converge on a focal point in about half of the trials, the Israeli players were able to coverage on a focal point in only about third of the trials. Interestingly, in a study conducted by Mehta et al. [3], it was found that the behavior of students with a British cultural background using the same type of game (game #9, Fig 2), was 0.29, even lower than the average ICA value obtained for the ICB group in our study (~0.33). This result is in agreement with the finding that the British culture is even more individualistic than the Israeli one; according to the Hofstede scale the British receive a score of 89 on the individualism scale, while the Israelis receive a score of 52. These results illustrate that the cultural background affects the level of coordination ability at the group level.

Another interesting observation concerns game #7, which is a fully symmetric game in which none of the three prominent rules was evident. Looking at Table 1, it can be seen that the coordination performance of the Chinese was not better than chance level (${\bar{ICA}}_{CCB}\approx 0.5$). In contrast, the coordination level of the Israeli group was much higher than chance level (${\bar{ICA}}_{ICB}\approx 0.75$). This result might be explained by the fact that in symmetric games Israelis tend to prefer items located in right spatial positions and regard them as focal points [23].

### The individual level of analysis

To examine the effect of the cultural background on focal point selection with even a higher resolution than specific game instances, we will now cluster the individual strategic profile within each cultural group. Recall that the strategic profile describes the propensity to choose each of the three available selection rules as part of the focal point selection process of each individual participant. For each individual player the strategic profile is composed of three SR values [Closeness, Equality, Accession], calculated on all 14 games, that define the specific location of each participant in a theoretical 3D strategic space.

**Fig 3:**
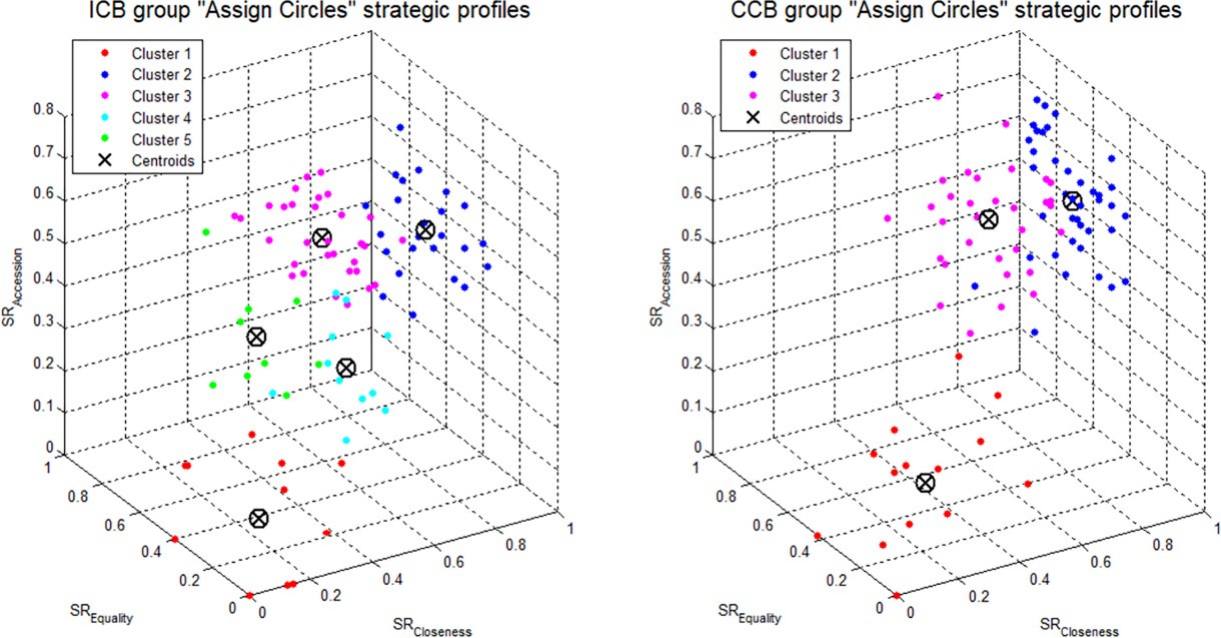
Clustering analysis results in 3D space.

We used K-Means clustering (e.g. [24,25]) to detect the typical strategic profiles for the ICB and the CCB groups. Fig 3 displays a visualization of the SR data of each participant from each of the cultural groups. Each point in the 3D space represents the three SR values of each candidate. In addition, the centroids of the different clusters are marked with a black "X". Each point in the 3D space could be assigned to its cluster by color.

The clustering results revealed that the CCB and ICB groups had a different number of clusters (number of strategic profiles): the data of the CCB group was divided into three clusters whereas the data of the ICB group into five clusters.

From examining the clustering results (Fig 3), several important insights can be drawn regarding the similarity and differences between the two groups. First, in both groups, a subset of the population of about the same relative size (~15%) selects its strategy randomly (orange dots, cluster #1). This sub-group is associated with a low coordination ability and the participants belonging to that cluster were not using any specific strategy. Second, we can see that in both groups there are two clusters, marked as cluster #2 and cluster #3 (covering similar locations in the 3D SR space), that represent players with a high coordination ability that show a preference for a specific strategy, either closeness or equality.

Table 2 presents the relative sizes of each of the clusters per group and the location of each of the clusters (in [x,y,z] coordinates) as well as the mean ($\bar{X}$) and standard deviation (σ) of the ICA scores associated with the cluster. It can be seen that while for the CCB group the behavior of all players can be summarized by the three clusters (clusters #1-#3) described above, the ICB group can be characterized by additional two clusters (clusters #4 and #5) not present in CCB, that together correspond to 23% of the players (Table 2). These two clusters contain players that are endowed with a mediocre coordination ability since they are inconsistent in their choice of a strategy.

**Table 2:** ICB and CCB clustering results.

Cluster # Number	Cluster description	ICB cluster location	ICB cluster relative size	CCB cluster location	CCB cluster relative size
#1	Low coordination ability	[0.15,0.20,0.08], $\bar{X}$ = 0.225, σ = 0.117	12.9%	[0.25,0.27.0.13], $\bar{X}$ = 0.240, σ = 0.007	16.8%
#2	High coordination ability: closeness priority	[0.94,0.61,0.47], $\bar{X}$ = 0.579, σ = 0.071	27.9%	[0.93,0.59,0.55], $\bar{X}$ = 0.574, σ = 0.057	47.3%
#3	High coordination ability: equality priority	[0.77,0.90,0.39], $\bar{X}$ = 0.619, σ = 0.046	36.5%	[0.80,0.84,0.45], $\bar{X}$ = 0.582, σ = 0.063	35.9%
#4	Medium coordination ability: closeness priority	[0.59,0.45,0.27], $\bar{X}$ = 0.397, σ = 0.073	11.9%	Does not exist	Does not exist
#5	Medium coordination ability: equality priority	[0.50,0.80,0.24], $\bar{X}$ = 0.444, σ = 0.051	10.8%	Does not exist	Does not exist

From the above results it is evident that the pattern of emerging clusters of CCB is contained within the pattern of ICB. This is because the ICB is associated with additional two clusters (#4, and #5, Fig 3) absent in case of CCB. The absence of these two clusters of mediocre coordination ability in case of CCB might suggest that the participants in that group, except for the low coordination ability participants, tend to converge to common strategies that lead to higher rates of successful coordination. Therefore, the identity of the CB is important for predicting the strategic behavior of players in the context of tacit coordination games. This will be demonstrated in the next section where we show that knowing the CB improves the accuracy of the predictive model.

The cluster analysis described above was based on all 14 games, the ten predefined and the four randomized games. We have chosen to include four extra randomized games since their inclusion leads to a greater variability in the data and consequently enables us to observe greater variability in strategic behavior. To verify that there are no differences in the clustering results between the larger and the smaller data sets (14 and 10 games, respectively) we have conducted an additional cluster analysis on the smaller set. Following this extra analysis we have obtained the same clustering results for each of the cultural groups. The results of the cluster analysis obtained for the smaller set appear in Supporting Information–S1 Appendix.

### The effect of the cultural background on predicting individual coordination ability (ICA)

In order to examine the effect of CB on the prediction of the ICA we have constructed two logistic regression models, one which does not use the CB as an input feature and the other that does. This was done in order to evaluate the contribution of the CB feature to the accuracy of ICA prediction.

For both models we used a logistic regression model with a split of 70%-30% for train and testing sets. Moreover, the randomized permutations for the sets were proportional to the cluster size of each group. The train group included 132 samples (65 ICB and 67 CCB), whereas the test group included 56 samples (28 of each CB). The best classifier out of 1000 iterations was the following:

$$ICA=0.09881+0.27059*SR_{Closeness}+0.28930*SR_{Equality}+0.09483*SR_{Acession}$$ (Eq 3.1)

$$R^{2}=0.89803,P<0.001$$ (Eq 3.2)

This classifier explained 89.8% of the variance in the train data. Running the test data in this model resulted in a mean absolute error of 0.038755 and in a median absolute error of 0.035013. This value reflects the difference between the predicted and actual ICA values in the training set.

Next, we constructed a similar logistic regression model, but this time we have utilized the CB feature by inserting a selector variable into the regression model, as follows:

$$ICA=CB(0.1037+0.2963*SR_{Closeness}+0.2251*SR_{Equality}+0.1205*SR_{Acession})+(1-CB)(0.0982+0.2488*SR_{Closeness}+0.3269*SR_{Equality}+0.0838*SR_{Acession})$$ (Eq 4.1)

$$R^{2}=0.90277,P<0.001$$ (Eq 4.2)

Since we have only two CB values a simple binary selector variable was defined to distinguish between the ICB and CCB training samples. When the value of the CB = 1, the equation reflects the model for the Chinese group, whereas when CB = 0 the equation reflects the model for the Israeli group. It can be seen that the explained variance has increased to 90.2%, the mean absolute error of the training set was 0.032706, and the median absolute error was 0.022267.

To quantify the improvement in prediction ability when utilizing the CB feature, we have computed the difference of the mean and absolute errors between the two models (with and without the CB feature):

$$mean\;absolute\;error\;improvment=\frac{\left|0.032706-0.038755\;\right|}{0.032706}=0.1850=18.5\%$$ (Eq 5.1)

$$median\;absolute\;error\;improvment=\frac{\left|0.022267-0.035013\;\right|}{0.022267}=0.5724=57.24\%$$ (Eq 5.2)

A considerable improvement can be seen in both measures reflecting the importance of including the CB feature in ICA predictive models. Noteworthily, these results were obtained although we have not used a pure random selection of samples to the train/test sets, but rather permutations that were proportional to the cluster size of each of the CB groups.

## Discussion

The purpose of this study was to examine the effect of the cultural background on focal point selection. To this end, individual strategic profiles were constructed and subsequently clustered. In addition, the contribution of the cultural background to the accurate prediction of coordination ability was assessed by logistic regression. The analysis at the group level has shown that the cultural background affects the level of coordination. That is, in our set of games, the Chinese appear to be better coordinators than the Israelis (see Table 1). This result may be associated with the classification of the Chinese culture as a collectivist society [18,19], while the Israelis are considered to be a more individualistic oriented society [11,16]. This explanation is corroborated by the results of the cluster analysis that was performed on the individual data points. Specifically, clustering of the ICA measure resulted in two different clustering profiles: whereas the Chinese could be characterized by two main strategies (i.e., closeness and accession), the Israelis could be characterized by four different strategies.

From Table 1 it is evident that the Chinese are better coordinators since they can more easily converge on the same solution when the set of options to choose from is smaller. That is, the Israelis are more versatile in their choice of a strategy whereas the Chinese utilize a more limited strategic space. This result is strengthened by findings from the literature emphasizing several factors affecting the diversity of the cultural strategic space. One of the factors concerns the role of homophily in human cooperation. Specifically, it has been shown that a higher homophily (the degree of homogeneity among members of a culture) is associated with a quicker convergence towards cooperation [26]. Therefore, it might be the case that a smaller set of strategies to choose from facilitates this quicker convergence, while a larger set of options decreases the probability to converge on the same solution. Another factor is strategic uncertainty. It has been suggested that in a cultural homogenous society players face less strategic uncertainty and therefore there is a greater chance for its members to converge on the same focal equilibrium [27]. That is, a more limited strategic space, associated with the Chinese group in our study, might be one of the mechanisms that diminishes strategic uncertainty. Lastly, it has been shown that collectivist cultures are more dependent on others in their decision-making process, and consequently, they might rely more on the support of others in their groups [8]. A greater chance to receive support for one’s own decision might occur when the set of options is depleted rather than diverse, as demonstrated by the Chinese group in this study.

From the clustering analysis (Fig 3) it can be seen that the CCB players operate in one of two distinct ways; they either do not implement any of the strategies at all (clusters #1 in Table 2), or alternatively, they adhere to one of two strategies (clusters #2 and #3 in Table 2). In contrast, in the ICB group there are two additional clusters, which are absent in the CCB group (clusters #4 and #5 in Table 2). These two clusters might reflect a more inconsistent behavior demonstrated by the players belonging to these clusters. That is, the coordination difficulty of a specific game might impact the chosen selection rule as follows. When it is easy to coordinate these players might use the prominent selection rule. However, when it is difficult to coordinate there is a lower probability of detecting a prominent rule and therefore the players decide randomly among the available selection rules.

Finally, to demonstrate the utility of the CB feature we constructed logistic regression models to predict the value of the individual coordination ability (ICA). We had two different logistic models, one which considers the CB feature and one which does not. Interestingly, the logistic regression analysis demonstrated that the effects of SR for closeness and accession were more pronounced in the CCB group than in the ICB group, but the effect of SR for equality was the opposite. This result might be explained by an effort to reduce strategic uncertainty by the CCB group. Specifically, accession and closeness rely on grouping principles, Gestalt principles of visual perception [28], whereas equality relies on higher order top-down processes influencing the fair division of resources. Thus, the reliance on Gestalt principles exploit visual organizational rules [29] that might consequently result in extracting similar meaningful representations of the board games across participants, whereas equality might entail a larger diversity in decision making.

Furthermore, it was shown that the model with the CB feature demonstrated a better accuracy as reflected in both the mean and median absolute error values. This result was obtained using a rather simple binary selector model while accounting for the proportion of the cluster size of each group. We believe that using a more complex learning algorithm (e.g. Artificial Neural Network) would result in an even better utilization of the CB feature and consequently in a higher predictive accuracy.

## Conclusions

The results of this study show that the cultural background has a profound effect on the strategic profile and on the ability to succeed in tacit coordination games. Moreover, the current study emphasizes the importance of relying on the individual level of analysis and not only on the group level of analysis. At the individual level of analysis a unique strategic profile was constructed for each individual player and the clustering of these individual profiles enabled us to gain further insights regarding the differences between the strategic spaces of each cultural group. In contrast, the analysis at the group level could not provide such a high in-depth resolution of the components of each of the strategic spaces and thus important insights regarding the strategic behavior associated with each cultural group would have been lost had we not reverted to the individual level of analysis. Consequently, the insights regarding the cultural background should be considered when constructing an automated agent that needs to coordinate with a human counterpart, since the inclusion of the CB feature substantially improves the predicative ability of the ICA.

## Future work

The results of the current study open a variety of new research directions. From the social and behavioral sciences it is well known that the cultural background also affects the social orientation value [16] of the individual as well as competitive and cooperative behaviors in group tasks [15,30]. Noteworthily, the higher coordination ability observed in the CCB group might be partly due to the unique construction of the games we used. Therefore, in future studies, in order to generalize the results, it would be interesting to include a larger and a different set of “Assign Circles” games. Moreover, it would be also worthwhile to explore tacit coordination games of different domains. Furthermore, it will be interesting to investigate the influence of the CB on the behavior of players in diverge interest tacit coordination games. In these games the reward scheme for the players is not symmetric and might result in unequal payoffs. In addition, it will be interesting to expand our research framework to explore additional cultures such as cultures at the extreme end of individualism, like the USA or UK (with Hofstede scores of 91, and 89 respectively), and observing whether it will be possible to extract a larger set of strategies (that is, more than five clusters).

## Supporting information

S1 Appendix Supporting information. (DOCX)

S1 File CSV Data files. (ZIP)
