# Supplementary material for: Collectivism-individualism: Strategic behavior in tacit coordination games
Source: PLoS One. 2020 Feb 4;15(2):e0226929. doi: 10.1371/journal.pone.0226929 (PMC6999890; doi:10.1371/journal.pone.0226929)
Supplement: S1 Appendix — (DOCX) [file pone.0226929.s001.docx]

**S1. “Assign Circles" predefined game boards**

The 10 predefined games are presented in Fig 4. The board's structure is based on the design of questions 11-20 in [3]. The order of appearance of the various games in the application was completely random.


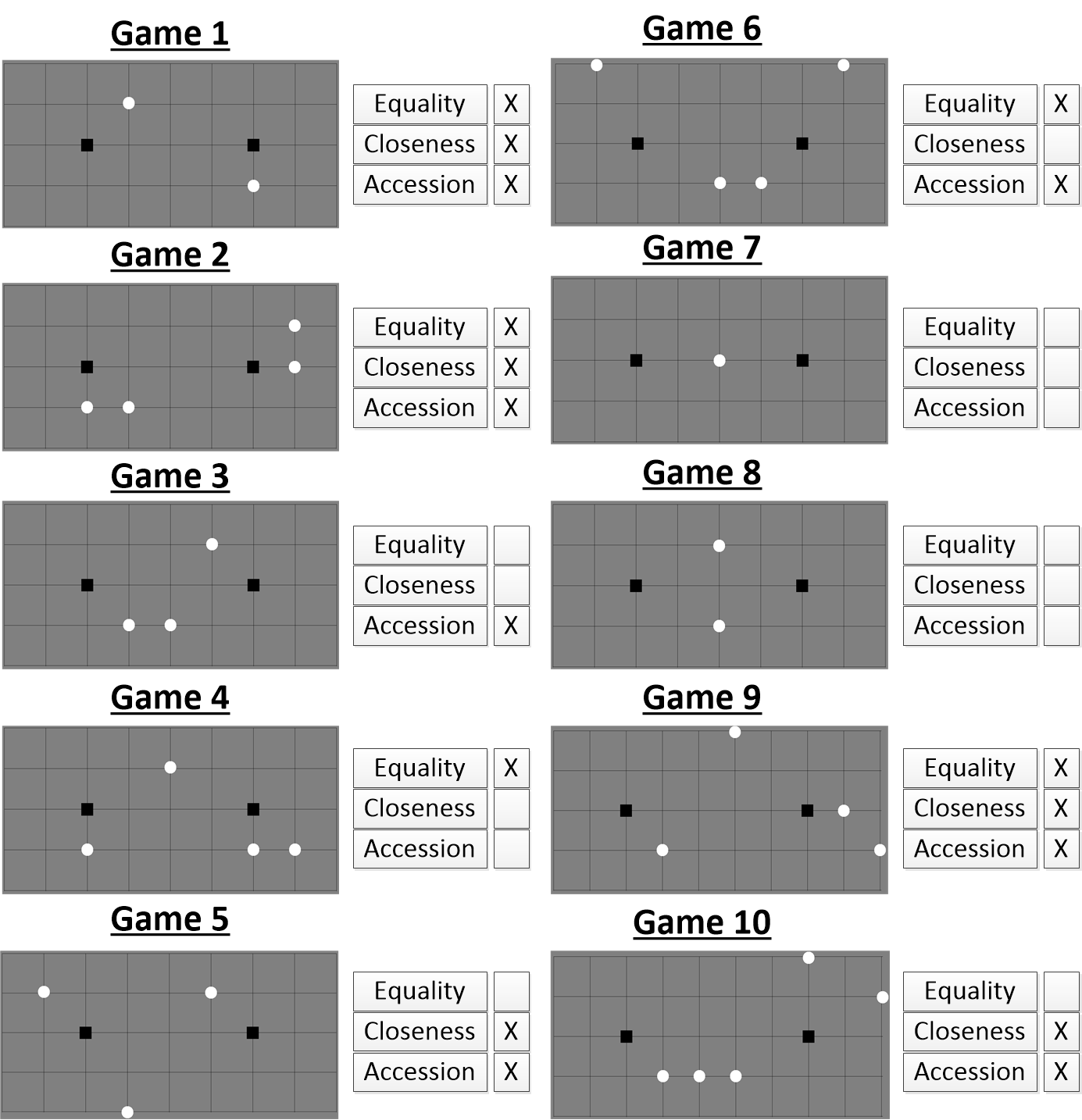


S1. Supporting Information.docx. A

**S2. "Assign Circles" experiment written instructions**

Given below are the written instructions that were presented to participants prior to the performance of the "Assign Circles" task. The written instructions followed the same set of instructions that were first delivered verbally.

"In this experiment, we wish to test your coordination ability against a random and unknown partner. You and your partner have a common goal, but you do not have the ability to communicate with each other.

In the following experiment, you are about to play in 14 games; in each game you must assign each circle to one of the presented squares, the left square or the right square. You should do this by dragging each circle via mouse until it intersects with one of the squares and then release it; the connection between the selected square and the center of the circle is marked by a straight white line.

For example, if there are two circles, you may connect each one of the circles to one square (one to the left square and one to the right square); alternatively, you may connect both circles to the right square, or you may connect both of them to the left square.

To score a point in each game, all circles in the game must be connected exactly in the same way as your counterpart who will be chosen randomly from the pool of subjects.

You can change your selections by dragging the circles again. Changes can be made multiple times until you click on the "next game" button that will take you to the next game. If you click the "previous game" button, you must make at least one circle drag until you will be enabled to click on the "next game" button. A button is enabled when it appears in green, whereas a button appearing in red is disabled, thus clicking on it will not affect the course of the game."

**S3. selection rules implementation in the predefined games (#1-#10)**

In this section we present the complete definition of the three selection rules. The following definitions are directly quoted from [3], pp. 173-175:

***Closeness:*** *For the rule of closeness to be applicable in a game, there has to be some commonly recognized concept of closeness of association between the two classes of objects (circles and squares). In the case of our grid, the most obvious and unambiguous measure of closeness of association between a circle and a square is the distance between them. Thus we interpret the rule of closeness as: assign each circle to the nearer square.*

***Accession:*** *The rule of accession implies that if a set of circles form a coherent group, all the circles in the group should be assigned to the same square. We shall say that two circles are connected if they are located at adjacent points in the grid, linked by a horizontal or vertical line, and we shell interpret a 'coherent group' as a set of connected circles. We define the distance between a square and a set of connected circles as the distance between the square and the nearest circles in the set. Then we interpret the rule of accessions the following formula: assign each set of connected circles to the nearer square.*

***Equality:*** *The rule of equality suggests the general formula: if there is an even number of circles, assign half of them to the one square and half of them to the other square. As stated, this rule never implies a unique assignment of circles to squares; we posit the median line rule as a subrule of refinement of the rule of equality, which uses the metric of closeness to discriminate among equal assignments. This rule is: if there is a vertical line such that an equal number of circles lie on each side, then assign circles left of the line to the left-hand square, and circles right of the line to the right hand square.*

Table S3. (taken from [3]) presents the specific solutions obtained by implementing each one of the three selection rules in games 1-10. Each one of the selection rules can only be implemented in a game board only if it defines a unique choice (there is only a single interpretation of the solution). In Table S3. “L” represents a connection of a circle to the left square and “R” to the right square. The circles are ordered from left to right and from top to bottom. For example, in game #3 the assignment implied by the rule of accession denoted by “LLR”, means that the two bottom circles are connected to the left square and that the upper circle is connected to the right square.

S1. Supporting Information.docx. B

| Game | Unique assignment implied by rule of | | | Predicted responses |
| --- | --- | --- | --- | --- |
|  | Closeness | Accession | Equality |  |
| 1 | LR | LR | LR | LR |
| 2 | LLRR | LLRR | LLRR | LLRR |
| 3 | None | LLR | None | LLR |
| 4 | None | None | LLRR | LLRR |
| 5 | LLR | LLR | None | None |
| 6 | None | LRRR | LLRR | LRRR or LLRR |
| 7 | None | None | None | None |
| 8 | None | None | None | None |
| 9 | LRRR | LRRR | LLRR | LRRR or LLRR |
| 10 | LLRRR | LLLRR | None | LLRRR or LLLRR |

**S4. clustering results for the set of the predefined games (#1-#10)**

This section includes the results of the cluster analysis conducted on the data set which includes only the ten predefined games. Table S4. presents the relative sizes of each of the clusters per group and the location of each of the clusters (in [x,y,z] coordinates) as well as the mean ($\bar{X}$) and standard deviation (σ) of the ICA scores associated with the cluster. It can be seen that while for the CCB group the behavior of all players can be summarized by the three clusters (clusters #1-#3) described above, the ICB group can be characterized by additional two clusters (clusters #4 and #5) not present in CCB, that together correspond to 20.4% of the players (Table S4.). These two clusters contain players that are endowed with a mediocre coordination ability since they are inconsistent in their choice of a strategy.

S1. Supporting Information.docx. C

| **Cluster #**  **Number** | **Cluster description** | **ICB cluster location** | **ICB cluster relative size** | **CCB cluster location** | **CCB cluster relative size** |
| --- | --- | --- | --- | --- | --- |
| #1 | Low coordination ability | [0.13,0.16,0.12],$\bar{X}$ = 0.209, σ = 0.109 | 11.8% | [0.26,0.32.0.22],$\bar{X}$ = 0.264, σ = 0.084 | 20.0% |
| #2 | High coordination ability: closeness priority | [0.95,0.68,0.67],$\bar{X}$ = 0.586, σ = 0.059 | 31.2% | [0.93,0.66,0.74],$\bar{X}$ = 0.587, σ = 0.049 | 58.9% |
| #3 | High coordination ability: equality priority | [0.71,0.97,0.52],$\bar{X}$ = 0.615, σ = 0.046 | 36.6% | [0.69,0.97,0.59],$\bar{X}$ = 0.578, σ = 0.040 | 21.1% |
| #4 | Medium coordination ability: closeness priority | [0.63,0.50,0.40],$\bar{X}$ = 0.424, σ = 0.081 | 12.9% | Does not exist | Does not exist |
| #5 | Medium coordination ability: equality priority | [0.34,0.77,0.22],$\bar{X}$ = 0.450, σ = 0.064 | 7.5% | Does not exist | Does not exist |

**S1 File. CSV Data files.zip**

**S1 File. CSV Data files.zip**
